# Exploring medical students’ perceptions of individual and group-based clinical reasoning with virtual patients: a qualitative study

**DOI:** 10.1186/s12909-024-05121-x

**Published:** 2024-02-25

**Authors:** Ipek Gonullu, Alper Bayazit, Sengul Erden

**Affiliations:** Faculty of Medicine, Department of Medical Education and Informatics, Ankara University, Cebeci, Ankara, Turkey

**Keywords:** Clinical reasoning, Individual vs. group-based, Virtual patient, Qualitative study, Medical students

## Abstract

**Background:**

Virtual Patients are computer-based simulations used to teach and evaluate patient interviews, medical diagnoses, and treatment of medical conditions. It helps develop clinical reasoning skills, especially in undergraduate medical education. This study aimed to and investigate the medical students’ perceptions of individual and group-based clinical reasoning and decision-making processes by using Virtual Patients.

**Methods:**

The study group comprised 24 third-year medical students. Body Interact^®^ software was utilized as a VP tool. The students’ readiness and the courses’ learning goals were considered when choosing the scenarios. Semi-structured interview forms were employed for data collection. MAXQDA 2020 qualitative analysis software was used to analyze the data. The students’ written answers were analyzed using content analysis.

**Results:**

The participants perceived individual applications as beneficial when making clinical decisions with Virtual Patients, but they suggested that group-based applications used with the same cases immediately following individual applications were a more appropriate decision-making method. The results indicated that students learn to make decisions through trial and error, based on software scoring priorities, or using clinical reasoning protocols.

**Conclusion:**

In group-based reasoning, the discussion-conciliation technique is utilized. The students stated that the individual decision-making was advantageous because it provided students with the freedom to make choices and the opportunity for self-evaluation. On the other hand, they stated that the group based decision-making process activated their prior knowledge, assisted in understanding misconceptions, and promoted information retention. Medical educators need to determine the most appropriate method when using Virtual Patients, which can be structured as individual and/or group applications depending on the competency sought.

**Supplementary Information:**

The online version contains supplementary material available at 10.1186/s12909-024-05121-x.

## Background

Virtual patients (VPs) are computer-based simulations used to teach and evaluate patient interviews, medical diagnoses, and treatment of medical condition [1–3]. VP activities can be designed based on individuals or groups, and their effectiveness depends on the activity design and not just on the virtual patients they employ [2]. VPs also instruct students in procedural and team skills [3], as well as help them develop communication, decision-making, situational awareness, leadership, and professionalism skills [4]. In terms of measurement and evaluation, they can be applied to both formative assessment of student performance and summative assessment of student success [5]. A study found that VP evaluations elicited similar results for those from clinical instructor evaluations and during the interactive learning process, VPs also can be employed to evaluate students’ reasoning and decision-making abilities objectively [6]. Unlike traditional simulation methodologies, VPs can generate continuous and predictable improvement in user performance through feedback and algorithms [7].

VPs contribute to the development of clinical reasoning skills, particularly in undergraduate medical education [8]. Clinical reasoning can be defined as skills, processes, or outcomes in which physicians observe, collect, and interpret data to diagnose and treat patients [9]. The process includes taking a medical history, performing a physical examination, confirming medical records, and providing a conclusive diagnosis. Clinical reasoning education aims to enable students to determine effectively which history-taking questions and examination methods should be used to make a correct diagnosis [10].

Gamification theory in education emphasizes illustrating goals and their relevance, nudging users through guided paths, giving users immediate feedback, and reinforcing good performance, which can offer an approach to enhance clinical reasoning [11]. Integrating clinical reasoning through case-based discussions and gamification creates a more engaging and interactive learning experience [12]. This integration starts by framing clinical scenarios as missions or challenges, creating game-like environments and interventions [13]. Points, levels, and badges are introduced to give users immediate feedback and a sense of accomplishment. The VP software, which provides real-time reactions, can be used to offer positive feedback, such as earning a badge when the virtual patient’s symptoms decrease or show improvement, indicated by the normalization of vital signs and improvement in breathing. This progress can motivate the learners, help track their progress, and improvement in learning performance [14]. Furthermore, gamification integrates aspects of storytelling and role-playing, allowing learners to take on the role of a healthcare professional navigating clinical cases. This immersive experience improves clinical reasoning and decision-making skills by applying theoretical knowledge to practical scenarios similar to real-world settings. Using gamification through individual clinical reasoning in medical education can foster an engaging and interactive learning experience. It can encourage self-directed learning and enhance the practical application of theoretical knowledge [15].

Collaborative clinical reasoning emphasizes the value of teamwork in achieving optimal clinical outcomes and patient safety [16]. Social Learning Theory (SLT) emphasizes the importance of collaborative interaction and observational participation [17]. It highlights learning through observation, imitation, and modeling, providing valuable insights into how collaborative learning can be optimized for educational purposes. During group-based clinical reasoning, the SLT emphasizes the significance of observational learning. Observing peers’ reasoning and decision-making processes allows students to learn from their experiences and the mistakes of others [18]. The theory highlights the importance of discussions and interactions in the learning process. This experience can enhance students’ understanding, activate prior learning, and help them identify critical queries for reaching a differential diagnosis. In addition, social learning also plays a crucial role in developing essential skills like communication, teamwork, and leadership. Individuals participating in small group discussions can share and discuss ideas. This process can enhance their communication skills and ability to express and understand diverse perspectives [19]. Group decision-making fosters teamwork as members coordinate, delegate, and support each other [20] to treat and cure virtual patients. Leadership training in medical education, methods like small group teaching, project-based learning, mentoring, and coaching were commonly employed [21], fostering the emergence of leadership skills as individuals learn to motivate, guide others, and assume responsibility in group settings.

As group-work has been an important context for self-regulation, it has been suggested that these regulatory processes could have an interpersonal level in group-work. The researchers consider that group cognition is the result of ‘aggregation’ of minds and the different individual cognitive systems interact to achieve the learning outcomes of group-work [22]. According to a group of researchers, self-regulated learning is an important predictor of socially shared regulation of learning and should be considered when designing small group activities and their environments [23]. Collaborative learning environments like small group-work can enable learners to engage in activities that are valuable for facilitating the learning process, like self-directed learning, justifications, and reflections or developing arguments [24]. Students can practice their clinical reasoning skills individually or in groups with VPs [25, 26]. Individually or as a group, VPs can be used for various purposes as an educational practice tool. For example, at the individual level, they might be used to determine the progression or order of a learning session [27, 28]. At the group level, they can be utilized to bring together individuals with diverse perspectives and abilities, and help them progress toward a shared goal [26]. Before selecting whether individual or group-based applications are appropriate for developing instructional activities with VPs, instructors must consider the student competency desired. By integrating both individual and group clinical reasoning processes in line with the VP scenario, learners can be aware of their knowledge and skill shortcomings, as well as gain the ability to make group decisions. Identifying student experiences in group and individual practices can guide medical educators and researchers.

## Methods

### Aim of the study

This qualitative study aimed toinvestigate the medical students’’ perceptions towards individual and group-based clinical reasoning and decision-making with VPs. The research questions were as follows:

1. How do the students view differently their experience of individual and group-based decision making in VP simulations?

2) How do the students approach group-based decision-making processes with VP simulations?

3) What do the students think about VP simulation and its contribution to their professionaldevelopment?

### Study setting

The “Clinical Reasoning with Virtual Patient” elective course is held one day each week for two hours. The course’s learning outcomes include patient-centered clinical evaluation and patient management, as well as arranging interventional procedures for diagnosis and/or treatment.

Based on the course’s objectives, after taking the course, students should be able to:

- Recognize the importance of basic life support for patients in the emergency department.
- Formulate a primary diagnosis based on findings from the anamnesis and physical examination.
- Select diagnostic tests to evaluate for pre-diagnoses.
- Formulate differential diagnoses by integrating anamnesis, physical examination, and diagnostic findings with physiopathology and clinical science knowledge.

**Fig. 1:**
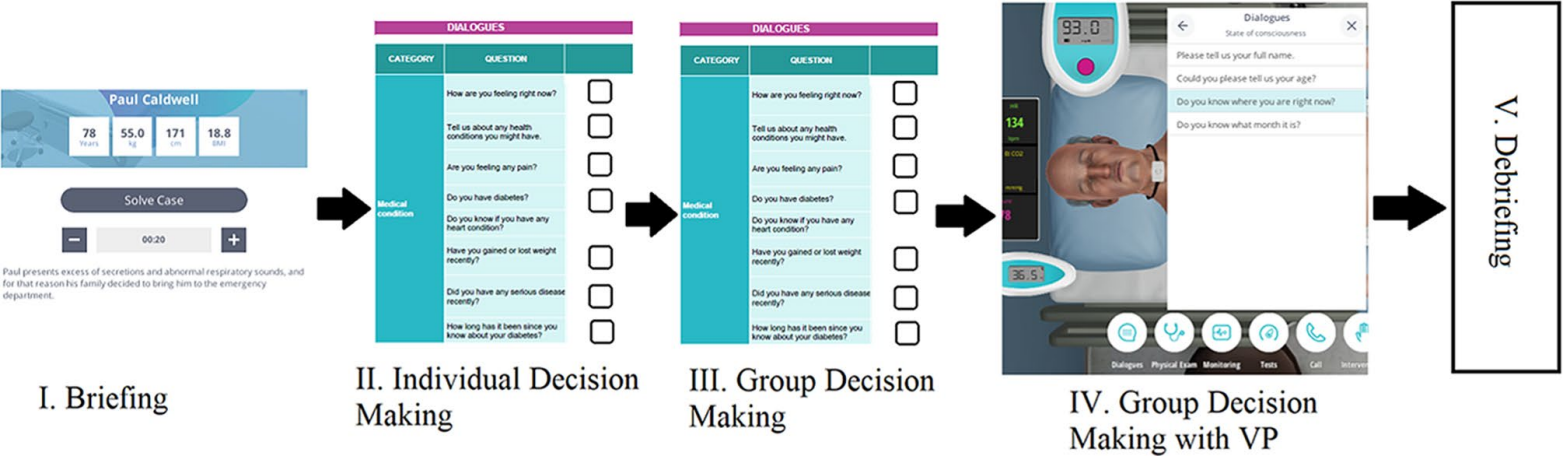
Application flow

During the sessions, Body Interact^®^ software was utilized as a VP tool. During the first session, students were informed about the lesson’s teaching methodology, the VP program, and its application. Furthermore, an explanation of the study was provided, and students’ written informed consent was collected. Applications were conducted in five stages (Fig. 1).

I. Briefing: A summary of the scenario and the following steps is provided.
II. Individual Decision Making: At this stage, students were given 10 min to think about the scenario and apply clinical reasoning procedures. Taking the Body Interact^®^ scripts into consideration, anamnesis, physical examination, and test questions were transformed into a checklist for the scenarios (Appendix 1). Students were asked to rank the anamnesis, physical examination, and test questions individually based on their clinical reasoning and priorities (1. must be done/asked/requested immediately, 2. can be done/asked/requested, or 3. not required at this time).
III. Group Decision Making: Students were assigned randomly to groups of three or four at this stage and instructed to complete the checklist form once again via group discussions. Students then managed their group dynamics and produced a final group decision through collaboration and clinical reasoning processes. They presented this conclusion as a collaborative decision on a single checklist form (the same as the second stage).
IV. Group Decision Making with a VP: At this stage, each group used the simulations to implement the group decisions they made. In the VP simulation, they performed priority query operations depending on the form they completed during the third stage, and the patient interface provided real-time interaction results (patient responses, physical examination responses, and test results).
V. Debriefing: Upon completion of the application, the groups’ software performance scores were displayed on-screen. After all groups practiced with the VP, the students were invited to verbalize their emotions and thoughts. The instructors then conducted group discussions regarding students’ incomplete and incorrect choices made during the application phase.

Some of the VP applications were completed face-to-face, while others were completed through distance education due to the COVID-19 pandemic. In this study, eight scenarios were used altogether, including two (one myocardial infarction case and one pneumonia case) in face-to-face education and six (five COVID-19 cases and one hypoglycemia case) in distance education. The students’ readiness and the courses’ learning goals were considered when choosing the scenarios.

While the courses were taught through distance education, the VP program was made available to students online. In this process, the scenario names, and forms to be entered were sent to the students, who were asked to complete the forms based on the scenarios and send them back. However, the students who could not make group decisions made only individual decisions.

### Virtual patient simulation

**Fig. 2:**
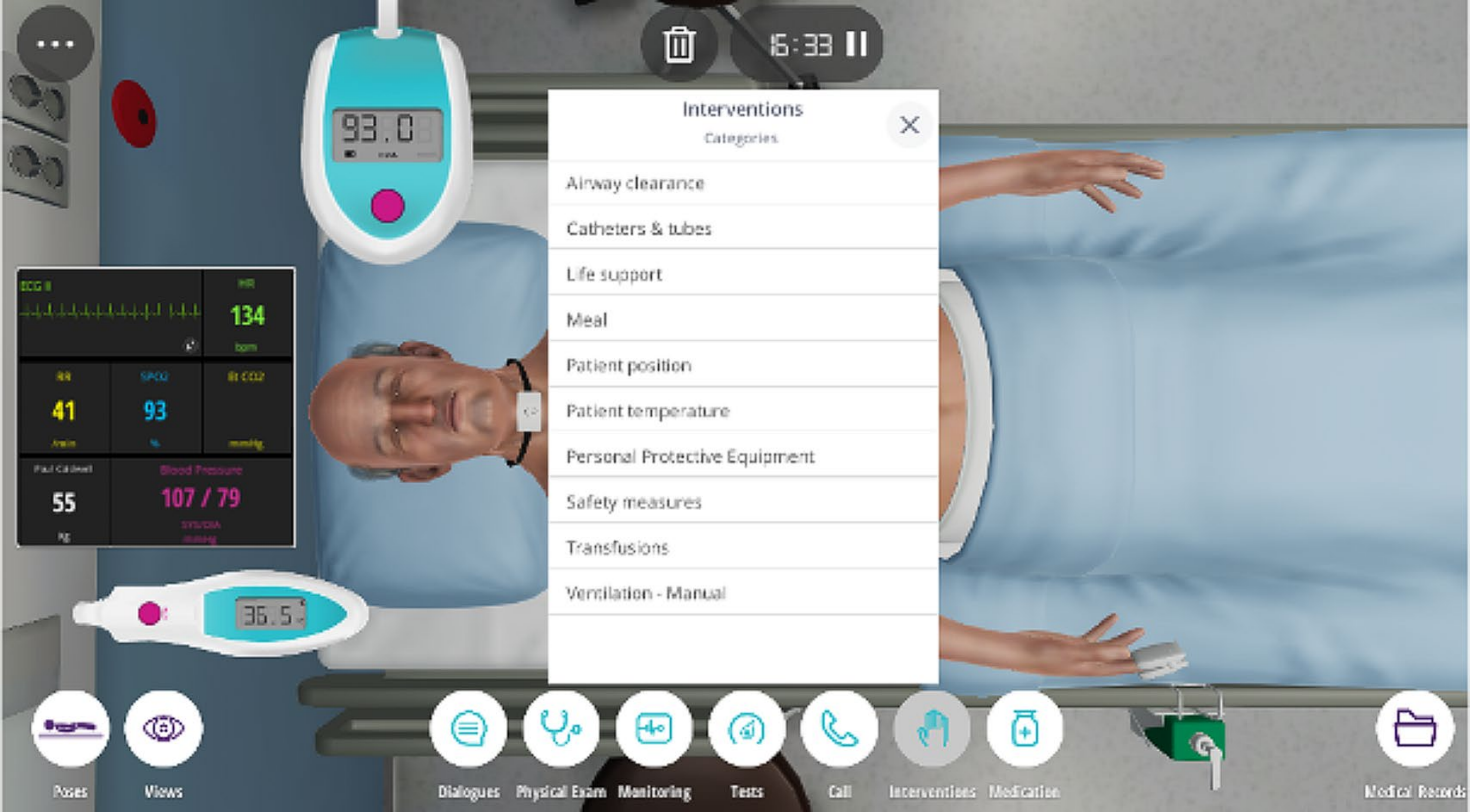
Body interact^®^ user interface

Body Interact^®^ is a platform that offers education and training through VPs that respond in real time to medical interventions (Fig. 2). When a case is completed or the simulation time is up, a dashboard with performance ratings is presented. These ratings are calculated based on the users’ interactions and queries. The software is available for PCs (with a desktop application), PDAs, the web (through browsers), and multi-touchscreen devices. In this study, we used both the PC and web-based (https://bodyinteract.com) versions during the data collection processes.

### Participants

Students were selected for the study through “criterion sampling,” which is one of the purposive sampling methods with 2019–2020 academic year Spring Semester and 3rd term pre-clinical medical students. The criteria for participation in the study comprised taking the “Clinical Reasoning with Virtual Patient” course and volunteering to participate in the study. When selection was completed, the sample comprised 24 students (10 females and 14 males).

### Data collection procedures

To collect data in the study, the researchers prepared a semi-structured interview form (Appendix 2), but due to the pandemic, which required that courses be held online, the interviews could not be conducted face-to-face, and the interview form was sent to the students via e-mail.

### Data analysis

MAXQDA 2020 qualitative analysis software was used to analyze the data. In this framework, students’ written answers were imported to the qualitative analysis software, and content analysis was used. During the content analysis phase, the students’ responses were read, and the first codes were revealed. The themes then were created from the codes and finalized by checking whether the themes and codes formed a suitable pattern. During the data analysis, three researchers coded the data set separately, then discussed the codes and themes to reach a consensus. Altogether, 68 coded structures and 440 code sections were created in 24 text documents.

## Results

### **RQ 1. 1** how do the students view differently their experience of individual and group-based decision making in VP simulations?

The analysis found that students have varying views on individual and group-based discussions in decision-making processes. Two themes were detected for the first research question.

### Theme 1.1: views on the individual decision-making process

The students reported positive aspects from the individual decision-making process, e.g., giving them the freedom to make decisions, observe consequences from individual mistakes, and conduct self-evaluations:

S21, M: I think it would be more useful if this application was individual and online rather than in groups. … I think that (the) online and individual application will be more beneficial for us because we see our own mistakes when we do it individually (sub-theme: observing consequences from individual mistakes).

S17, F: Individually, we learn to trust our own knowledge and opinions (sub-theme: conducting self-evaluations).

### Theme 1.2: views on integrating processes

The students stated that in the decision-making process with VP scenarios, integrating the process by first applying it individually, then through group discussion, made them conscious of being team members, as well as recognizing their faulty or incomplete information, thereby bringing different views together on common ground. Furthermore, they said that this approach supports active learning, provides new knowledge and perspective, and increases learning permanence. However, they also stated that this approach can cause difficulties in communication and decision-making processes:

S3, M: … I think it reinforces the team-thinking approaches and my spirit (as) being (part of) a team (sub-theme: making them conscious of being team members).

S11, M: In group decisions, everyone has a different approach to a subject, and a more comprehensive decision is made by combining these approaches. In individual activity, these comprehensive issues may be disregarded (sub-theme: bringing different views together on common ground).

S19, F: I can say that discussing and brainstorming with my group friends after individual decisions gave me different perspectives. It also helped me remember things I didn’t know or forgot (sub-theme: providing new knowledge and perspective; recognizing their faulty or incomplete information).

S7, M: … With individual and group decision-making formats being integrated, we first questioned ourselves and revealed our information as much as possible, then different information from friends…. I think it provides more careful and active learning (sub-theme: supporting active learning).

S21, M: We could not communicate well as a group, so everyone’s decisions could not be considered. All my friends experienced clinical reasoning but remained passive because they could not express themselves. Likewise, sometimes I couldn’t make the group accept the decisions I wanted (sub-theme: causing difficulties in communication and decision-making processes).

To sum up, while the students found individual applications useful in clinical decision-making processes with VPs, they stated that integration of the processes (group discussions for the same scenario immediately after the individual decision-making) provided a more useful decision-making process.

### **RQ 2.2** how do the students approach group-based decision-making processes with VP simulations?

It was observed that some students changed some of their individual decisions after group discussions. Three themes emerged from this research question.

### Theme 2.1: approaches in the individual decision-making process

It was determined that the students developed different approaches in individual decision-making processes with the VP: (1) trial and error; (2) using the VP software’s scoring priorities; and/or (3) following the clinical reasoning steps:

S21, M: Due (to) my weaknesses in the treatment phase, I made more progress through trial and error (sub-theme: trial error).

S11, M: After a few uses, I was able to comprehend the simulation’s expectations (of) me, and I fulfilled its requirements (sub-theme: using the VP software’s scoring priorities).

S14, M: In the first applications, I gave priority to questions and physical examinations directly related to the patient’s complaints. However, when I saw that in some scenarios, unrelated situations also affected the treatment, I expanded my questions and examinations in the next scenarios (sub-theme: following the clinical reasoning steps).

S15, F: I have always done these choices in an orderly manner, first, I took anamnesis, and I acted according to which question I should ask first. I did the same in physical examination, tests, medications, and diagnosis, respectively (sub-theme: following the clinical reasoning steps).

### Theme 2.2: approaches in decision-making processes with the group

The students employed two basic approaches when deciding through group discussion: (1) exchanging ideas and agreeing on the information they view as correct, (2) following predetermined steps:

S3, M: In fact, we applied the reasoning and decision-making processes that I applied individually in a similar way with the group. We determined the diagnosis, necessary tests, (and) findings by brainstorming. Listening to other ideas in the group, we agreed on these methods and applied them in the same way (sub-theme: exchanging ideas and agreeing on the information they view as correct).

S14, M: We realized that we selected too many queries when in the individual decision-making phase. When we came together as a group, we tried to prioritize the more necessary ones. We tried to (reach) a common decision by taking everyone’s opinion on every question and every item. (With) the items where we selected different items, we tried to learn from each other what we did not know (sub-theme: exchanging ideas and agreeing on the information they view as correct).

S7, M: I can say that we have an approximate standard for anamnesis and physical examination. … we reached a synthesis result by approaching different information and ideas rationally and questioningly in diagnosis and treatment (sub-theme: following predetermined steps).

### Theme 2.3: reasons for changing decisions as a result of group discussion

The reasons why students changed their decisions from the individual process after participating in-group discussions included: (1) awareness of lack of knowledge and misconceptions; (2) awareness of lack of knowledge about the scenario; (3) agreeing with their peers’ arguments; and (4) considering the software’s scoring priorities. They were found to receive:

S16, F: The explanations and views of my group mates regarding their decisions did make sense to me. It brought to my attention several details I had ignored. I also gained knowledge in topics I did not know well. These reasons caused me to change my mind (sub-theme: awareness of lack of knowledge and misconceptions).

S8, M: After explaining my decision, as a result of the discussion, the more logical and grounded ideas of my group mates were enough to change my decision. For example, I learned that glucagon should be given to a hypoglycemic patient with diabetes (sub-theme: awareness of lack of knowledge and misconceptions; agreeing with their peers’ arguments).

S14, M: Another reason is that we changed it by considering (to) which criteria the system might have given priority. For example, maybe I will not apply the Glasgow Coma Scale in real applications, but we changed it by considering that the system probably wants it (sub-theme: considering the software’s scoring priorities).

To sum up, students followed clinical reasoning steps in VP applications, but some students who gained experience with different scenarios using the software noted its scoring features, and then made decisions based on its priorities. During group discussions, students changed their minds by noticing their misconceptions or lack of information.

### **RQ 3. 3** what do the students think about VP simulation and its contribution to their professionaldevelopment?

The students stated that VP applications helped them in many ways, which were grouped under three themes.

### Theme 3.1: contributing to cognitive domain

The students stated that decision-making processes with VPs: (1) ensured the permanence of what has been learned; (2) provided clinical reasoning skills; (3) provided the ability to interpret test results; and (4) provided an opportunity to learn medication:

S3, M: In my opinion, it is an application that is similar to PBL lessons, but thanks to simulation, it is much more memorable, realistic, and we can see the results of our mistakes. When we give the interventions to a real patient, we can realize the results of our mistakes, and we can learn the right decisions (sub-theme: ensuring the permanence of what has been learned).

S19, F: … The persistence of observing the results of my interventions during the right or wrong implementation process. When I think about it, I was able to put a few things in my mind that I heard in the theoretical lessons, but could not understand, thanks to these applications (sub-theme: ensuring the permanence of what has been learned).

S8, M: I found it very successful, especially in terms of learning clinical reasoning, and therefore, I strongly recommend it to be used in medical school courses (providing clinical reasoning skills).

S13, M: During the treatment process, I think that as I apply more to the work, I give more normal doses of medication (providing an opportunity to learn medication).

### Theme 3.2: contributing to affective domain

Students stated that VP practices helped them by (1) reducing the level of anxiety toward clinical applications, (2) increasing their motivation and professional self-confidence, and (3) helping them deal with feelings of panic, particularly in emergency cases:

S14, M: Interventions in emergencies seemed difficult and complex to me. I realized that thanks to this simulation, it became easier as I practiced. I can say that I have partially overcome my fear…. I can say that seeing the reflection of the information we have learned in the clinic has increased my motivation toward the lessons, partially (sub-theme: reducing the level of anxiety toward clinical applications).

S10, M: The virtual patient application made a significant contribution to the increase (in) my self-confidence. Taking responsibility by calmly responding to an emergency or life-threatening patient (situations) increases self-confidence and enables us to be prepared for such situations in the future (sub-theme: increasing their motivation and professional self-confidence).

S19, F: I think it helps us to learn to control our panic, excitement, and stress…. I tried to keep calm to save the patient as much as I could in sudden situations (sub-theme: helping them deal with feelings of panic, particularly in emergency cases).

### Theme 3.3: contributing to gaining clinical experience

Medical students stated that they can gained a sense of clinical experience in decision-making processes through VPs, allowing them to realize the difficulties experienced in clinical practice, as well as learn from their mistakes:

S3, M: The virtual patient simulation has given me familiarity on how I should approach patients before I pass to clinical phases of my education (sub-theme: gaining sense of clinical experience in decision-making processes).

S8, M: Being able to practice is very important; it is very easy to access this opportunity thanks to the virtual patient application (sub-theme: gaining sense of clinical experience in decision-making processes).

S4, F: In short, we have taken a lesson that is a risk-free simulation of our professional life for us and what we have lived has remained a sweet experience for all of us (sub-theme: gaining sense of clinical experience in decision-making processes).

S18, M: Since there are no actual patients, we can act and learn with high flexibility. It makes us aware of the gaps in our knowledge (sub-theme: learning from their mistakes).

In summary, the decision-making processes used during the VP simulation led preclinical students to appreciate its professional benefits. They found that it allowed them to experience the feeling of interacting with a real patient without the risk of harming one. Table 1 summarizes the subthemes for individual and group-based clinical reasoning according to our results.

**Table 1:** Sub-themes obtain form student answers

	Individual clinical reasoning	Individual & group clinical reasoning
Positive aspects	giving them the freedom to make decisions	making them conscious of being team members
	observe consequences from individual mistakes	recognizing their faulty or incomplete information
	conduct self-evaluations	bringing different views together on common ground
		supporting active learning
		providing new knowledge and perspective
		increasing learning permanence
Negative aspect	N/A	causing difficulties in communication and decision-making processes

## Discussion

This study investigated students’ perceptions in clinical reasoning processes with VPs and their views on the contribution of this practice to their professional development. With the first research question, in which they were asked for their opinions on individual and group-discussion decision-making processes, the students stated that the individual decision process was useful in terms of allowing the freedom to decide, demonstrating the consequences of mistakes, and providing opportunities for self-evaluation. While a single participant is sufficient for computer-based educational tasks that do not require divided attention because they do not require interpersonal coordination, groups of two or three are more successful than an individual participant as task complexity increases [29]. Accordingly, the number of people in the group can be determined by the task’s complexity, and in some cases, individual practices may be more beneficial than group practices. This can be explained by the ease of VP scenario levels in students’ positive perception towards individual clinical reasoning. However, group practices were beneficial in terms of getting feedback from students and gaining new ideas to improve their clinical performance, but students viewed talking about mistakes in these groups negatively [30]. This can be viewed as another factor affecting the opinions of the students who participated in our study and found the individual VP assessment more beneficial than group discussions.

Some students stated that group discussions held immediately after individual decision making provided a more useful decision-making process. Conducting group discussions during sessions with VPs to retain information [31]. As a result of students sharing their perspectives, observations, and prior experiences while completing the application in a group, the information can be reanalyzed, reformed, and restructured. SLT suggests that people can learn new information and behaviors by watching others. In our study, students engaged in group discussions after individual decision-making, which allowed them to observe their peers’ perspectives, observations, and experiences [17]. Some students who participated in our study also supported this view and stated that they gained new knowledge and perspective through group discussions, and that the permanence of what was learned increased. SLT posits that social interaction plays a critical role in the learning process. Through group discussions, students actively engage with it, discussing and sharing insights, a key component of learning in social contexts [26].

With the second research question, students’ clinical reasoning processes were discussed. It has been determined that they manage the process through trial and error, consider software scoring priorities, and/or follow clinical reasoning steps in clinical decision-making processes. Students feel more comfortable trying different strategies in a gamified setting without fear of real-world consequences. Case-based discussion and gamification strategies engage [12] students and foster a trial-and-error approach, where students can learn from their mistakes and refine their clinical reasoning skills towards a common goal [11]. For instance, in our study, students made an incorrect diagnosis or intervention, and the virtual patient provided instant feedback such as dizziness, bruising, and sweating, allowing the student to understand their error and try a different approach. On the other hand, students with moderate self-regulation skills in particular can demonstrate gaming-system behavior [32], which can be explained by the fact that learners try to be successful or get a high score in an educational environment by utilizing the help, results, or feedback features of the system instead of trying to learn the material [33]. In this study, instead of transforming students’ theoretical knowledge into practice processes through clinical reasoning steps, the choice to progress by considering trial-and-error or software-scoring priorities can be viewed as “gaming the system.”

The students reached common decisions by exchanging ideas in their approaches to the decision-making process with the group, and they followed the clinical reasoning steps. Decisions that individuals made alone in decision-making processes were investigated with decisions that individuals made through group discussion-conciliation [20]. The results indicated that the students who used group discussion-conciliation made more accurate decisions than those who employed individual decision-making. In our study, students reported that they used group discussion-conciliation processes while making decisions with the group in VP applications, and that their decisions were more accurate compared with the individual decision-making process. Another study found that using VPs in a collaborative learning activity was more effective in improving students’ knowledge and retaining treatment decisions than in an independent learning activity [25].

Another finding that emerged from the study was that after using the individual approach, students sometimes changed their decisions after working with a group. It has been determined that being aware of theoretical or scenario-knowledge deficiencies, accepting their peers’ ideas, or considering software scoring priorities affect these decision changes.

With the third research question, which examined VPs’ contribution to professional development, we found that these practices benefitted students in terms of gaining cognitive, affective, and clinical experience. Among these contributions, VPs provided learning permanence, boosted clinical reasoning skills, provided experience in treatment processes, reduced anxiety levels, increased motivation and professional self-confidence, and created a sense of treating real patients without the risk of harming them. These results supported the outcomes of previous studies [6, 8, 34–36]. Although VPs elicited little effect on knowledge acquisition, VP users prepared themselves for clinical experience and viewed them as a good resource to help them reinforce their skills [37].

This was a qualitative study in which 24 medical school students participated, with the results limited to those who participated. Furthermore, the sessions that initially were held face-to-face had to move online due to the COVID-19 pandemic; therefore, the applications were made individually in the online course process, and the decision-making process could not be realized with the group. Thus, students’ group decision-making experiences comprised face-to-face sessions, another limitation of the study.

## Conclusions

VP is a simulation method that provides an opportunity to evaluate the stages – e.g., data collection, diagnosis, and patient management – used in the clinical reasoning process, monitor changes in student performance, and provide feedback. The effective use of VPs in medical education plays an essential role in achieving learning goals and permanence in learned knowledge. For this reason, medical educators must determine the most appropriate method when using VPs, which can be structured as individual and/or group applications depending on the competency sought. The students perceived that the individual decision-making process is beneficial in terms of giving freedom to make decisions, as well as the opportunity for self-evaluation. They also stated that the group decision-making process can be beneficial in terms of activating students’ prior knowledge, as well as helping them recognize knowledge deficits and gain learning permanence. Based on the research findings, the following are nine tips for educators:

1. Facilitate Post-Individual Decision-Making Discussions: After individual clinical decision-making, encourage group discussions to enable students to share information and reanalyze it, leading to more informed and refined decisions.
2. Integrate Group Discussions with VP Sessions: During virtual patient sessions, incorporate group discussions to improve retention and practical application.
3. Encourage Sharing of Student Perspectives: Promote a comfortable environment where students can share observations and prior experiences, improve their communication skills, and exchange information.
4. Focus on Clinical Reasoning Processes: Guide students to effectively manage clinical reasoning processes through structured steps and trial and error to make decisions.
5. Address “Gaming the System” Behavior: Be aware that students are not demonstrating gaming the system behavior. Guide them towards focusing on the clinical reasoning process rather than simply aiming for high scores.
6. Promote Group Discussion-Conciliation: Encourage group discussion and conciliation to achieve shared decisions, leading to more accurate outcomes.
7. Acknowledge the Role of Peer Influence and Knowledge Gaps: It’s important to acknowledge that students may change their decisions after group discussions due to the awareness of knowledge gaps or the influence of peers’ ideas. Peer influence can play a crucial role in the learning process.
8. Emphasize VPs’ Contribution to Professional Development: Highlight how working with VPs contributes to cognitive, affective, and clinical experience, enhancing skills like clinical reasoning, reducing anxiety, and increasing motivation and professional confidence.
9. Use VPs for Realistic Practice: Utilizing VPs provides a low-risk way for students to gain experience in treatment processes and prepare for real clinical situations while reinforcing their skills in a safe environment.

In future studies, to support the findings and identify the difficulties experienced in these processes, a detailed examination will be made by including retrospective thinking, in-group interaction metrics, and lecturer observation reports.

### Electronic supplementary material

Below is the link to the electronic supplementary material.

**Appendix 1:** Scenario Checklist

**Appendix 2:** Semi-Structured Interview Form

## Data Availability

The datasets generated during and/or analysed during the current study are available from the corresponding author on reasonable request.

## Abbreviations

VP: Virtual Patient
SLT: Social Learning Theory
