# Supplementary material for: Exploring medical students’ perceptions of individual and group-based clinical reasoning with virtual patients: a qualitative study
Source: BMC Med Educ. 2024 Feb 25;24:189. doi: 10.1186/s12909-024-05121-x (PMC10895817; doi:10.1186/s12909-024-05121-x)
Supplement: Supplementary file 1 — Appendix 1: Scenario Checklist [file 12909_2024_5121_MOESM1_ESM.docx]

**Appendix-1: Scenario Checklist**

**According to the scenario, evaluate the following items and categorize them between 1 and 3 in terms of their appropriateness for this scenario.**

**1:** must be done/asked/requested, **2:** can be done/asked/requested, **3:** not necessary

**NAME&SURNAME / GROUP NAME:**

The patient is male, sixty-six years old, and diabetic. He suffers from shortness of breath and seems discomforted due to unknown reasons.

**History Taking***

|  | **QUESTIONS/REQUESTS** | **NECESSITY (1/2/3)** |
| --- | --- | --- |
| Medical Conditions | How are you feeling right now? |  |
|  | How long have you been feeling like this? |  |
|  | Have you felt like this before? |  |
|  | Tell us about any health conditions you might have. |  |
|  | For how long do you have diabetes? |  |
|  | Are you feeling any pain? |  |
|  | Have you gained or lost weight recently? |  |
|  | Did you have any serious disease recently? |  |
|  | How much do you weigh? |  |
| Medication | Are you taking any medication? |  |
|  | Have you felt any side effects from your medication? |  |
| Nutrition | What do you usually eat? |  |
|  | When was the last time that you had something to eat? |  |
|  | What was the last thing you ate? |  |
|  | Do you eat snacks in between meals? |  |
|  | Are you taking any supplements? |  |
|  | Have you felt any appetite changes? |  |
|  | Do you drink alcoholic beverages frequently? |  |

* The given items constitute only a part of the history-taking inquiries. In addition, ABCDE Assessment, Diagnostic queries were also asked to the students. For detailed information about the queries, please see the Body Interact^®^ instructor guidelines.
