# Supplementary material for: Exploring medical students’ perceptions of individual and group-based clinical reasoning with virtual patients: a qualitative study
Source: BMC Med Educ. 2024 Feb 25;24:189. doi: 10.1186/s12909-024-05121-x (PMC10895817; doi:10.1186/s12909-024-05121-x)
Supplement: Supplementary file 2 — Appendix 2: Semi-Structured Interview Form [file 12909_2024_5121_MOESM2_ESM.docx]

**Appendix-2: Semi-Structured Interview Form**

Demographic Data:

Age:

Gender:

Class:

Have you ever failed the class?

Interview Questions:

I would like to discuss your thoughts and opinions about the “Clinical Reasoning with Virtual Patient” course. I can give you time to review and reflect on all the practices we've done so far. You can ask me if there's anything you don't understand or want clarification on.

Can you tell us the first things that come to mind about the “Clinical Reasoning with Virtual Patient” course? (Content, format, process, trainers, participants, etc.)

1. What are the positive aspects of virtual patient applications?

2. What are the negative aspects of virtual patient applications?

3. What are your views on the simulation tool?

4. What are your views on the contribution of the virtual patient application to your professional development?

5. What are your suggestions for the use of virtual patient applications in medical school courses?

6. During the virtual patient practice, what strategy did you follow in your reasoning and decision-making process?

7. During the virtual patient application, what strategy did you follow in your reasoning and decision-making process?
